# Hepatitis C virus infection in EU/EEA and United Kingdom prisons: opportunities and challenges for action

**DOI:** 10.1186/s12889-020-09515-6

**Published:** 2020-11-09

**Authors:** Aya Olivia Nakitanda, Linda Montanari, Lara Tavoschi, Antons Mozalevskis, Erika Duffell

**Affiliations:** 1 Present address: Centre for Pharmacoepidemiology, Department of Medicine Solna, Karolinska Institutet, Stockholm, Sweden; 2 European Centre for Disease Prevention and Control, Stockholm, Sweden; 3 European Monitoring Centre for Drugs and Drug Addiction, Lisbon, Portugal; 4 Department of translational research and new technologies in medicine and surgery, University of Pisa, Pisa, Italy; 5 World Health Organization Regional Office for Europe, Copenhagen, Denmark

**Keywords:** Hepatitis C virus, Prisons, Injecting drug use, European Union, European Economic Area

## Abstract

**Background:**

Hepatitis C virus (HCV) transmission in the European Union, European Economic Area and United Kingdom is driven by injecting drug use (IDU), which contributes to the high burden of chronic infection among people in prisons. This study aimed to describe the context, epidemiology and response targeting HCV in prisons across the region.

**Methods:**

We retrieved and collated HCV-related data from the World Health Organization’s Health in Prisons European Database and the European Centre for Disease Prevention and Control’s hepatitis C prevalence database. Prisons population data were obtained from the Council of Europe Annual Penal Statistics on prison populations (SPACE I).

**Results:**

There were 12 to 93,266 people in prisons, with rates of 31·5 to 234·9 per 100,000 population. Median age was between 31 and 40 years, with up to 72% foreign nationals. Average detention time ranged from one to 31 months. Ministries of Health had sole authority over prisons health, budget administration and funding in 27, 31 and 8% of 26 reporting countries, respectively. Seroprevalence of HCV antibodies ranged from 2·3% to 82·6% while viraemic infections ranged from 5·7% to 8·2%, where reported. Up to 25·8 and 44% reported current and ever IDU, respectively. Eight countries routinely offered HCV screening on an opt-out basis. Needle and syringe programmes were available in three countries. Among the nine countries with data, the annual number of those who had completed HCV treatment ranged between one and 1215 people in prisons.

**Conclusions:**

HCV burden in prisons remains high, amidst suboptimal levels of interventions. Systematic monitoring at both local and regional levels is warranted, to advance progress towards the elimination of HCV in the region.

## Background

The advent of highly effective direct-acting antivirals (DAAs) and the launch of the Global Health Sector Strategy (GHSS) on viral hepatitis 2016–2021 [1] have boosted worldwide efforts targeting hepatitis C virus (HCV) considerably in recent years. However, about 1·75 million people still continue to be newly infected annually, and deaths attributable to viral hepatitis are increasing [2]. The GHSS aspires for 90% case detection and 80% treatment by 2030 [1]. Of the 71 million people living with chronic HCV infection globally, only 14 million were estimated to have been diagnosed in 2015 and only 1 million had accessed DAAs [2]. This highlights the need to find the ‘missing’ millions of people living with HCV infection, particularly those who are disproportionately affected [3] yet largely remain undiagnosed and untreated.

Injecting drug use (IDU) accounts for almost one quarter of the global HCV incidence, and 8% of those with chronic HCV infection currently inject drugs [2, 4]. Across the European Union, European Economic Area (EU/EEA) and United Kingdom, between 0.02 and 0.9% of the population are known to inject drugs and IDU is the key driver of the HCV epidemic [5]. While 3·9 million people across the EU/EEA and United Kingdom were estimated to be living with chronic HCV infection in 2015 [6], latest data indicate that where transmission was known, IDU was responsible for more than half of new cases [7]. The prevalence of antibodies against HCV (anti-HCV) across EU/EEA countries and United Kingdom ranges up to 5.9% [6] in the general population, but reaches 84% among people who inject drugs (PWID) [8]. Among the at-risk groups, similar estimates are observed among people in prisons as for PWID [8, 9].

Prior IDU is common among people in prisons, and a proportion continue to inject while in prison [10]. About 17% of the 590,000 people incarcerated in Europe on any single day are drug offenders [11]. Those convicted for drug-related crimes maybe disproportionately at risk of bloodborne infections like HCV [10]. In addition to IDU, transmission of blood-borne viruses (BBVs) in prison settings is facilitated by potentially high risk behaviours including sexual activity, tattooing and piercing [12] with needle-syringe and equipment sharing. Considering a median detention period of eight months coupled with high recidivism across European prisons [11], HCV acquisition and transmission is further exacerbated [13]. It is estimated that up to one in four people in prisons cross the EU/EEA has been exposed to HCV infection [14], and rises to over 60% among those with a history of IDU [15]. If untreated in prison, the persisting infection risk [13] remains a pertinent threat to public health as people in prisons return to communities upon release.

Although people in prison have been identified as a priority population, PWIDs are often excluded from conventional care as they sometimes face marginalisation and discrimination within communities [15, 16]. The European Centre for Disease Prevention and Control (ECDC) and the European Monitoring Centre for Drugs and Drug Addiction (EMCDDA) propose active case finding with early diagnosis of HCV infection in prisons as a strategy for prevention and entry into care pathways [17]. Harm reduction encompasses interventions aimed at preventing and reducing the negative outcomes caused by IDU: education and information; needle and syringe programmes (NSP); and opioid substitution therapy (OST) with methadone or buprenorphine for the management of opioid dependence; and have been found to be feasible for implementation in prisons. Further, DAA therapy is highly effective, even among PWID, those with human immune deficiency virus (HIV)/HCV co-infection and individuals on OST [15].

Overall, interventions in most countries have yet to be scaled up sufficiently to achieve the targets set in the Action plan for the health sector response to viral hepatitis in the World Health Organization (WHO) European Region (2017) [18] and hence the GHSS [19]. It is estimated that by reducing HCV transmission among PWID populations alone, more than 40% of new infections globally can be offset in the coming years [4], and specifically targeting those in prisons enables community dividends beyond this setting [20]. Because a proportion of people in prisons have experience with drug use, may continue or develop drug problems while incarcerated [21], interventions targeting IDU in prisons have the potential to improve both health and offending behaviours that lead to incarceration [22].

The potential impact of prisons-based interventions targeting HCV necessitates a better understanding of the opportunities and challenges in this setting, to advance progress towards elimination of HCV by 2030. This study aimed to describe the current context, epidemiological situation and responses targeting the prevention and control of HCV infection in prisons across the EU/EEA and United Kingdom.

## Methods

### Study design

A retrospective analysis of data submitted to the WHO‘s Health in Prisons European Database (HIPED) [23] by EU/EEA countries and United Kingdom.

### Data and data sources

Data collected through the national questionnaire for the minimum public health dataset for prisons in the WHO European Region in 2016/2017 for EU/EEA countries and United Kingdom were considered, and extracted from HIPED database on 4^th^ March 2020. These data were provided by national focal points from the relevant public authority(ies) responsible for prison healthcare services in each participating Member State and included public health indicators ranging from prison population statistics, the prison healthcare system, prison risk factors, disease screening and treatment of communicable diseases, among others [24]. Seroprevalences were based on anti-HCV and HCV ribonuclueic acid (HCV-RNA). Where not specified, the reference year for data collected was not available because the information was not requested in the original questionnaire. Data validation involved clarifying discrepancies and missing values with the national focal points [24].

Supplementary data on prisons populations were obtained from the SPACE I-2018 – Council of Europe Annual Penal Statistics: Prison populations [11]. The term ‘prisons’ is widely used in Europe to refer to all penitentiary institutions including those for sentenced individuals and those awaiting sentence, as drawn from this project.

Other data on HCV prevalence in prisons were also obtained from ECDC’s online prevalence database of infectious diseases [25]. The database contains peer reviewed literature on studies reporting on the prevalence of HCV published between 2005 and 2017, collated and appraised through a systematic review [26]. HCV prevalence data was downloaded from the ECDC database on 4^th^ March 2020 in CSV format, and only included measures of anti-HCV.

The detailed methodology of these three projects have been published elsewhere [11, 24, 25].

### Data analysis

Data management and basic descriptive analyses were performed using Microsoft Excel (2016). Maps were constructed using the ECDC Map Maker, EMMa [27].

## Results

The HIPED contained data from 26 countries including Germany which reported subnational data. There were no data for Austria, Greece, Hungary, Liechtenstein and Luxembourg.

### Prisons healthcare oversight

Healthcare oversight was described in terms of state institutions charged with the prisons healthcare authority, budget administration and funding. Twenty-six countries reported on the three parameters (Table 1).

**Table 1:** Prisons healthcare oversight: authority, budget administration and funding source, EU/EEA and United Kingdom (UK), 2016/2017

	Prisons healthcare authority	Prisons healthcare budget administration	Prisons healthcare funding source
Belgium	Ministry of Justice	Ministry of Justice	Ministry of Justice
Bulgaria	Ministry of Justice	Ministry of Justice	Ministry of Justice
Croatia	Ministry of Justice	Ministry of Justice	Health insurance and State budget
Cyprus	Ministry of Health	Ministry of Health	..
Czech Republic	Ministries of Health, Justice, Interior and Healthcare department of prisons systems	Ministry of Justice	Health insurance
Denmark	Ministry of Health and Healthcare Department of prisons systems	Healthcare department of prisons systems	Ministries of Health and Justice
Estonia	Ministry of Justice*	Ministry of Justice	Ministry of Justice
Finland	Ministry of Health	Ministry of Health	Ministry of Health and State budget
France	Ministry of Health	Ministry of Health	Ministry of Health and State budget
Germany	Ministry of Justice	**	**
Iceland	Ministries of Health, Justice, Interior and Healthcare department of prisons systems	Ministry of Health and Healthcare department of prisons systems	State budget
Ireland	Ministry of Justice	Healthcare department of prisons systems	Ministry of Justice
Italy	Ministry of Health	Ministry of Health	Ministry of Health
Latvia	Ministry of Justice	Ministry of Justice	State budget
Lithuania	Ministries of Health and Justice	Ministries of Health and Justice	State budget
Malta	Ministries of Health and Interior	Ministry of Interior and Healthcare department of prisons systems	Ministry of Health
Netherlands	Ministry of Justice	Ministry of Justice	Ministry of Justice
Norway	Ministry of Health	Ministry of Health	State budget
Poland	Ministry of Justice	Ministry of Justice	Ministry of Justice
Portugal	Ministry of Justice	Ministry of Justice	Ministry of Justice
Romania	Healthcare department of prisons	Healthcare department of prisons system and Health insurance	Ministries of Justice, Interior and Health insurance
Slovakia	Ministries of Health and Justice	Ministry of Justice and Health insurance	Ministry of Justice and Health insurance
Slovenia	Ministry of Health	Ministry of Health	State budget
Spain	Ministry of Health***	Ministry of Health***	Ministry of Health***
Sweden	Ministry of Justice	Ministry of Justice	Ministries of Health and Justice
UK	Ministry of Health	Ministry of Health	Ministry of Justice and State budget

*Source: HIPED*

*.. No data*

*Authority reported to be Ministry of Health in EMCDDA database [28]

**Prisons healthcare budget administration: Ministry of Justice and health insurance of individuals: 2 (North Rhine-Westphalia and Hesse), Ministry of Justice: 6 (Lower Saxony, Rhineland-Palatinate, Saarland, Berlin, Hamburg, Schleswig-Holstein), Ministry of Justice and state budget: 7 (Saxony, Saxony-Anhalt, Thuringia, Mecklenburg-Western Pomerania, Brandenburg, Bremen, Bavaria), State budget: 1 (Baden-Wüttemberg); Prisons healthcare funding source: Ministry of Justice: 15; Other: 1 (Brandenburg – Prisons)

***Ministry of Health in Catalonia and Ministry of Interior in rest of Spain

In 19 countries, a single institution had overall authority of the healthcare in prisons i.e. Ministries of Health (seven countries), Justice (eleven countries) or the healthcare department of prisons (one country); while joint authority was reported in the remaining seven countries. Eighteen reporting countries had their prison healthcare budget administered by a single institution, of which Ministries of Health were solely responsible in eight countries. In nine countries, it was administered by two or more institutions. On healthcare funding in prisons, the highest number (seven) had prisons health funded by the Ministry of Justice only and five through the state budget only. The Ministry of Health was the funding source in two countries, health insurance in one, while nine countries reported a combination of two or more funding sources. In Germany, the Ministry of Justice had overall authority across all 16 federal states, but varied between states for budget administration and funding.

### Prisons population

Data on prisons population were available for 28 EU/EEA countries and United Kingdom (Table 2). Belgium, Hungary and Malta did not participate in the survey. The total number of people in prisons in these countries ranged from 12 in Liechtenstein to 93,266 in United Kingdom. The prison population rate was lowest in Liechtenstein (31·5 per 100,000) and highest in Lithuania (234·9 per 100,000). Across these countries, their median age was between 31 and 40 years, and were incarcerated for an average 1·2 months in Cyprus to 31·1 months in Portugal. Most countries (17) reported average lengths of imprisonment of less than one year with eight less than six months, but in nine of 28 countries the mean length of imprisonment was more than a year, and exceeded two years in two countries. The proportions of female and foreign people in prisons ranged from 0 to 9·8% and 1·1 to 72.1%, respectively. In three EU/EEA countries: Austria, Greece and Luxembourg, the proportion of foreign people in prisons was over 50%.

**Table 2:** Characteristics of the prisons’ population, EU/EEA and United Kingdom (UK), as of 31 January 2018

	Total number	Prison population, per 100,000	Median age, years	Female, %	Foreigners, %	Average length of imprisonment, months
Austria	8960	101·6	34	5·8	54·7	9·3
Bulgaria	6988	99·1	..	3·1	2·7	13·1
Croatia	3190	77·7	37·2	4·6	8·9	4·7
Cyprus	643	74·4	43	6·7	39·7	1·2
Czech Republic	22,159	208·8	32·5	7·4	8·2	24·0
Denmark	3653	63·2	36	4·5	28·9	3·9
Estonia	2525	191·4	36	5·2	9·1	16·5
Finland	2815	51·1	35·6	7·3	17·5	6·3
France	69,596	103·5	31·9	3·6	22·1	8·7
Germany	64,193	77·5	..	5·8	38·1	7·7
Greece	10,036	93·5	..	5·5	52·7	12·4
Iceland	163	46·8	31	9·8	23·9	13·5
Ireland	3844	79·5	33	4	13·1	4·9
Italy	58,087	96	40	4·2	34·1	14·5
Latvia	3765	194·6	..	7·7	2·5	..
Liechtenstein	12	31·5	39	0	75	2·0
Lithuania	6599	234·9	39	5	1·7	10·7
Luxembourg	684	113·6	35	5·4	72·1	8·1
Netherlands	9315	54·4	35	5·1	19·5	3·5
Norway	3461	65·4	33	6·4	32·1	4·9
Poland	73,822	194·4	35	3·9	1·1	11·0
Portugal	13,440	130·6	..	6·4	16	31·1
Romania	23,050	118·1	35	4·7	1·2	24·2
Slovakia	10,028	184·2	..	7·1	2·2	13·6
Slovenia	1346	65·1	..	6·0	14·0	7·2
Spain	59,129	126·7	39	7·4	28	21·4
Sweden	5713	56·5	34	6·1	28·5	8·1
UK England & Wales	84,373	142·4	34	4·6	11·1	7·2
UK Northern Ireland	1453	77·0	32·7	4·4	8·7	4·2
UK Scotland	7440	136·5	..	4·5	..	..

*Source: SPACE I-2018 – Council of Europe Annual Penal Statistics: Prison populations* [11]

*.. Not available*

*All prison population numbers presented here refer to the non-adjusted figures, which includes those in non-penal institutions* i.e. *police stations, juvenile institutions, migrant detention centres, psychiatric institutions etc*

### HCV prevalence

Sixteen countries had national and/or subnational data on anti-HCV or HCV-RNA seroprevalence in the HIPED or ECDC prevalence databases (Table 3). The reporting period covered was from 2001 to 2016, with seven countries reporting data after 2014. The prevalence of anti-HCV ranged from 2·3% in a maximum-security psychiatric prison in Broadmoor, United Kingdom to 82·6% from two prisons in Berlin, Germany. HCV-RNA positivity ranged from 5·7% in Malta to 82% in England. Spain reported national-level data for 2000-2009, during which period the anti-HCV prevalence declined from 44·9 to 25·3% and further to 18.7% (2016).

**Table 3:** Prevalence of anti-HCV and HCV-RNA in prisons, EU/EEA and United Kingdom (UK)

	Year	Geographical coverage	Reference group	anti-HCV			HCV-RNA	
				Tested %	Tested *N*	Positive %	Tested %	Positive %
Bulgaria	2009	Unspecified (One prison and juvenile institution)	Adults +children	NA	498	24·7	..	..
	2010	Unspecified (2 juvenile institutions)	Children only	NA	258	20·5	..	..
Croatia	2007	National (All prisons)	Adults only	NA	3348	12·5	..	..
	2007	National (All juvenile institutions)	Children only	NA	140	4·3	..	..
	2006	Unspecified (Multicentre)	Adults only	NA	3348	14·2	..	..
Estonia	2015*	National	All (including those in remand prison/jail)	86·3	NA	41·1	..	..
Finland	2006	National (All prisons and juvenile institutions)	Adults +children	NA	384	45·8	..	..
	2006*		Sentenced only	..	..	42·3	..	..
France	2003	National	Unspecified	NA	31,215	6·8	..	..
	2010	National	Adults only	NA	1876	4·8	..	..
	2010	Southeastern France (3 prisons)	Adults only	NA	5957	5·2	..	..
	2013	Unspecified (5 prisons)	Adults only	NA	1720	6·5	..	..
	2013	Clermont-Ferrand and Riom (2 prisons)	Adults only	NA	342	4·7	..	..
	2003	Caen	Adults only	NA	442	3·9	..	..
Germany	2001	Berlin (2 prisons)	Unspecified	NA	174	82·8	..	..
	2002	Unspecified (1 young offenders institution)	Adults only	NA	1125	8·6	..	..
Hungary	2009	National (20 prisons)	Adults only	NA	4894	4·9	..	..
Ireland	2011	National	Adults only	NA	777	12·9	..	..
Italy	2002	Naples	Adults only	NA	524	37·4	..	..
	2002	Unspecified (Multicentre)	Adults only	NA	973	38·0	..	..
Malta	2017*	National	All (including those in remand prison/jail)	..	..	..	75	5.7
Portugal	2008	Coimbra (1 regional prison)	Adults only	NA	151	34·4	..	..
	2005	Unspecified (2 prisons)	Adults only	NA	445	10·78	..	..
	2015*	National	All (including those in remand prison/jail)	100	NA	14·4	..	..
Slovakia	2015*	National	All (including those in remand prison/jail)	17·5	NA	14·2	..	..
Spain	2000	National	Adults +children	..	..	44·9	..	..
	2001	National	Adults +children	..	..	42·9	..	..
	2002	National	Adults +children	..	..	38·9	..	..
	2003	National	Adults +children	..	..	37·8	..	..
	2004	National	Adults +children	..	..	37·2	..	..
	2005	National	Adults +children	..	..	33·0	..	..
	2006	National	Adults +children	..	..	30·0	..	..
	2007	National	Adults +children	..	..	29·0	..	..
	2008	National	Adults +children	..	..	27·0	..	..
	2009	National	Adults +children	..	..	25·3	..	..
	2008	18 prisons across Asturias, Cantabria, Lerida, Salamanca, Barcelona, La Coruna, Alicante.	Adults only	..	..	22·7	..	..
	2009	Valencia	Adults only	NA	2332	14·7	..	..
	2001	Alicante	Adults only	NA	730	38·2	..	..
	2016*	National	All (including those in remand prison/jail)	80 5	NA	18·7	66	60
Sweden	2015*	National	All (including those in remand prison/jail)	90	NA	35·0	90	35
UK	2011	Scotland (All prisons including juvenile institutions)	Adults +children	NA	4810	19·2	..	..
	2011	Oxfordshire (1 prison)	Unspecified	NA	118	11·0	..	..
	2012	Broadmoor (1 maximum security psychiatric hospital/prison)	Unspecified	NA	129	2·3	..	..
	2013	London (1 prison)	Unspecified	NA	511	4·3	..	..
	2016*	England	All (including those in remand prison/jail)	24	NA	18·0	33	82
	2016*	Northern Ireland	All (including those in remand prison/jail)	..	..	12	..	..
	2015*	Wales	All (including those in remand prison/jail)	13	..	6	..	..

Sources: ECDC Prevalence database for infectious diseases (Year denotes final year of sampling in study) and HIPED*

*.. No data; NA Not applicable*

### HCV infection screening

**Fig. 1:**
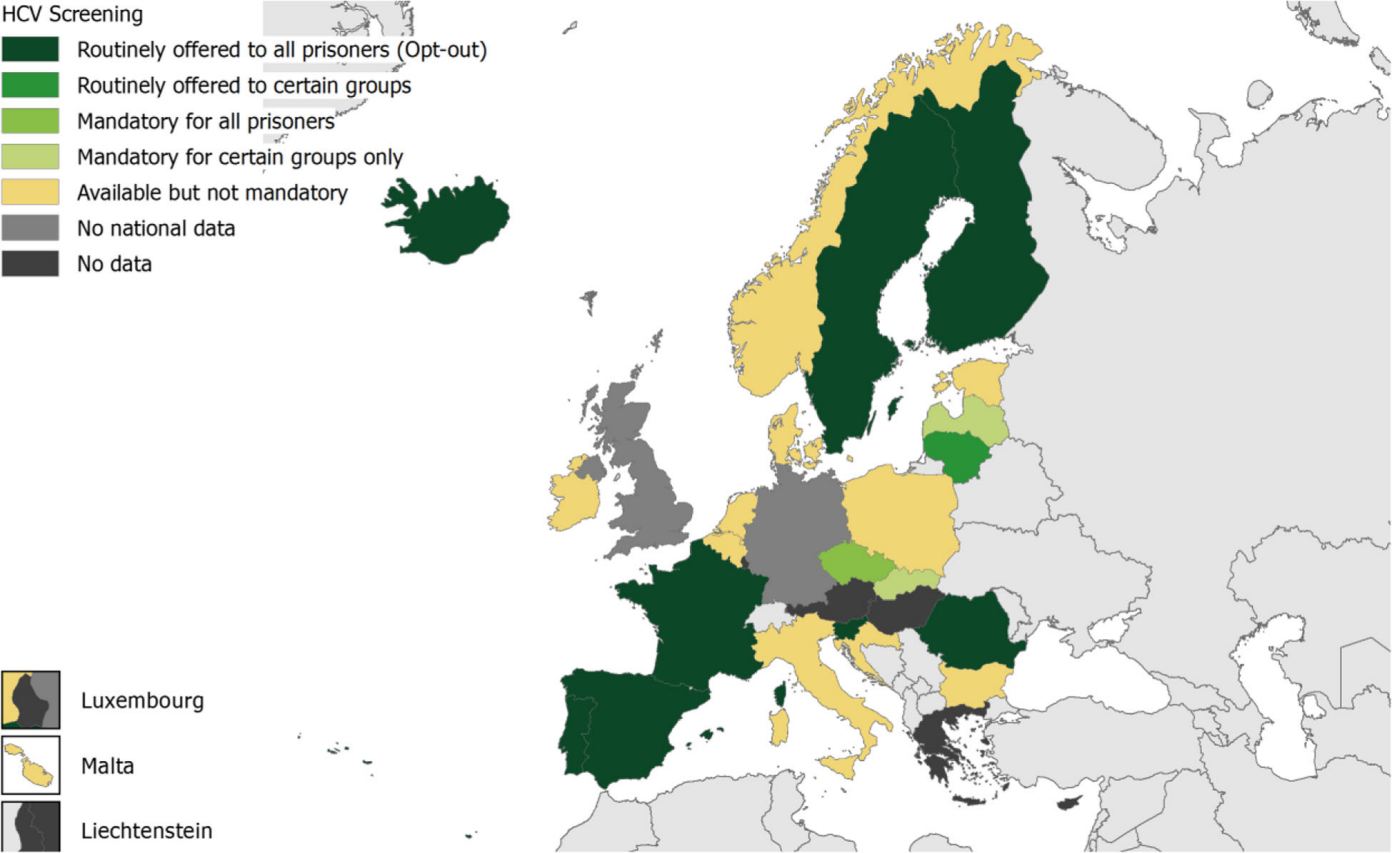
HCV screening in prisons, EU/EEA and United Kingdom (UK), 2016/2017. Source: HIPED

Twenty-five countries reported having HCV screening for people in prisons (Fig. 1). In 13 of these countries, testing was available but not mandatory and eight countries routinely offered testing to all people in prisons on an opt-out basis. In three countries: Latvia, Lithuania and Slovakia; testing was targeted at certain risk groups only. While such risk-based testing were offered on clinical suspicion in Lithuania, HCV screening was reported to be mandatory for those HIV positive in Latvia; and for drug users, sex offenders, drug dealers and foreign nationals in Slovakia. In the Czech Republic, testing was reported as mandatory for all people in prisons. Germany and United Kingdom reported on HCV screening at subnational level. HCV testing was not mandatory in 15 of the 16 German federal states. Of these, seven routinely offered screening to those eligible on an opt-out basis, was reported as ‘available’ in another seven and testing was done at the person’s request in one state. In Bavaria only, HCV testing was mandatory for all people in prisons. In the United Kingdom, England and Wales offered tests routinely to all on an opt-out basis, it was available but not mandatory in Northern Ireland, while Scotland offered when clinically appropriate. Cyprus did not report on HCV screening.

### IDU

Of the 26 reporting countries, seven had data relating to numbers of current and ever injecting drug users in prisons (Table 4). Only Italy, Romania, Slovakia and Spain had national-level data relating to all people in prisons. The prevalence of current injectors ranged from 0·06% in Northern Ireland (United Kingdom) prisons to 25·8% in Italy. Among countries with data on the proportion of people in prisons who had ever injected drugs, this ranged between 0·7% and 44·0%.

**Table 4:** Numbers and prevalence of current and ever injecting people in prisons, EU/EEA and United Kingdom (UK)

	Year	Current		Ever	
		n	%	n	%
Bulgaria*°*	2016	..	..	576	..
Croatia^	2015	..	..	28	0·7
Italy*	2014	13,465	25·8	16,712	32·0
Romania*	2015	..	..	21	..
Slovakia*	2015	408	4·5	..	..
Spain*	2015	3251	5·0	28,617	44·0
UK England*°*	2016	7163	18·0	13,958	35·0
UK Northern Ireland*°*	2016	76	0·1	..	..

*Source: HIPED*

*.. No data*

Reference population: *All people in prisons including those in remand prison/jail, ^Sentenced people only, °Missing

*For the United Kingdom only, ever injectors were explicitly defined as current injectors + previous injectors*

### Needles/syringe exchange programmes and opioid substitution treatment

Among the 26 countries with data on the availability of NSPs in prisons, only Spain reported it was available in all prisons (Table 5). Germany reported NSP exclusively in one wing of Berlin’s women prison.

**Table 5:** NSPs and OST in prisons, EU/EEA and United Kingdom (UK)

	NSPs	Eligibility for OST	Year	People on OST, n
Belgium	Unavailable	Sentenced and pre-trial detention	2016	686
Bulgaria	Unavailable	Sentenced only	2016	14
Croatia	Unavailable	Sentenced only	2016	414
Cyprus	Unavailable	Sentenced and pre-trial detention	2016	8
Czech Republic	Unavailable	Sentenced only	2015	53
Denmark	Unavailable	Sentenced and pre-trial detention	NA	..
Estonia	Unavailable	Sentenced and pre-trial detention	2016	71
Finland	Unavailable	Sentenced and pre-trial detention	2015	411
France	Unavailable	Sentenced and pre-trial detention	2015	5325
Germany	Available*	Sentenced and pre-trial detention	NA^2^	2780^2^
Iceland	Unavailable	Sentenced and pre-trial detention	..	4
Ireland	Unavailable	Sentenced and pre-trial detention	2015	479
Italy	Unavailable	Sentenced and pre-trial detention	2014	1647
Latvia	Unavailable	Sentenced and pre-trial detention	2015	50
Lithuania	Unavailable	Unknown^1^	NA	..
Malta	Unavailable	Sentenced and pre-trial detention	NA	..
Netherlands	Unavailable	Sentenced and pre-trial detention	NA	..
Norway	Unavailable	Sentenced and pre-trial detention	2015	409
Poland	Unavailable	Sentenced and pre-trial detention	2015	140
Portugal	Unavailable**	Sentenced and pre-trial detention	2015	1137
Romania	Available**	Sentenced only	2016	42
Slovakia	Unavailable	NA	NA	NA
Slovenia	Unavailable	Sentenced and pre-trial detention	NA	..
Spain	All prisons	Sentenced and pre-trial detention	2016	3532
Sweden	Unavailable	Sentenced and pre-trial detention	2016	50
UK-England	Unavailable	Sentenced and pre-trial detention	2015	29,146
UK-Northern Ireland	..	..	2016	38
UK-Scotland	..	..	2016	1711
UK-Wales	..	..	NA	..

*Source: HIPED*

*.. No data, NA Not applicable*

* Reports to EMCDDA indicate NSP available in one female prison [28, 29]

** Reports to EMCDDA indicate that projects to introduce NSP in Portugal and Romania formally approved but never fully implemented and not routinely available [28, 29]

^1^*Availability of OST in Lithuania is based on 2018 data* [28, 29] *not available in the HIPED*

^*2*^*Sum of numbers of inmates on OST in each federal state: 14 (North Rhine-Westphalia, reference date 30/04/2016); 318 (Hesse, 01/10/2016); 1 (Saxony 18/01/2017); 40 (Saxony-Anhalt 31/03/2016), 37 (Saxony-Anhalt 31/03/2016). The figures collected refer to the total number of substitution treatments, not to a specific opioid addiction; 31 (Thuringia 31/12/2016); 60 (Rhineland-Palatinate 31/03/2016), 2 (Mecklenburg-Western Pomerania 26/01/2017); 800 (Baden-Württemberg 2016); 100 (Bremen 28/01/2017); 2 (Saarland 20/01/2017); 35 (Bavaria 31/01/2016); 1068 (Berlin 2016); 150 (Hamburg 31/01/2017); 122 (Schleswig-Holstein 01/02/2017)*

Twenty-three of the 26 reporting countries had national-level data on OST. For the United Kingdom, eligibility for OST was only reported for England (Table 5). In 20 countries including all federal states of Germany, both sentenced and those in pre-trial detention were eligible for OST. In four countries, only sentenced individuals were eligible. The number of people on OST ranged from four in Iceland (year missing) to 29,146 in England (2015). There was no data relating to the number of people in prisons who were eligible for OST in the HIPED.

### Hepatitis C treatment

Nine of the 26 reporting countries had national-level data on HCV treatment completion in prisons (Table 6). During the 2015–2017 period, the number of people in prisons who had completed treatment ranged from 1 in Croatia to 1215 in Spain.

**Table 6:** People in prisons on HCV treatment, EU/EEA

	Year	People who completed HCV treatment, n
Belgium	2017	4
Croatia	2015	1
Czech Republic	2015	555
Estonia	2016	66
Latvia	2016	5
Portugal	2015	99
Romania	2015	71
Slovakia	2016	57
Spain	2017	1215

*Source: HIPED*

## Discussion

Prisons across the EU/EEA and United Kingdom have a disproportionately high prevalence of chronic HCV infection, owing to specific environmental, socioeconomic and healthcare associated risk factors within and outside of prison settings. This study aimed to collate and describe relevant context, epidemiology and response across EU/EEA prisons based on publicly available data, to better understand the opportunities and challenges in this setting as the region seeks to accelerate progress towards the GHSS targets and thus elimination of HCV by 2030.

The data highlighted significant variations between the administrative structures of prisons. Only Italy reported having a single centralised system with authority, budget administration and funding instituted by the Ministry of Health only. Similarly, six countries: Belgium, Bulgaria, Estonia, Netherlands, Poland and Portugal, had these functions solely overseen by the Ministry of Justice. Combined, the majority of EU/EEA countries (> 70%) have at least two institutions responsible for the oversight of healthcare in prisons. Although the WHO recommends prison healthcare to be under the remit of the Ministry of Health [30], these findings emphasise the need for multisectoral collaborations at the high level to scaling up any interventions in this setting, owing to the multisectoral governance of prisons healthcare.

We also found that people in prisons across the EU/EEA are diverse, with marked inter-country differences. Migrants and PWID comprise a notable proportion of the prison population in some countries, providing an opportunity in prisons to reach them with interventions that may not otherwise be as accessible in the community. In a few countries, the average detention period is long, extending over two years, which increases the risk of HCV acquisition and transmission among people in prisons engaged in high risk activities whilst imprisoned. At the same time, a longer detention period accommodates the care pathway for chronic HCV disease as the standard DAA treatment duration is now around 8–12 weeks. Such duration also allows for the uptake of OST.

There is still a lack of recent data, or even any data on HCV prevalence from about half of EU/EEA countries. Data from the ECDC database originated from an updated systematic review [8], complemented by more recent HIPED data. Owing to the lack of consistent data over the years, it was not possible to look into temporal trends for most countries, although national-level data from Spain showed a decline in anti-HCV seroprevalence from 45% in 2000 to 25% in 2009. However, the data presented clearly indicate that the burden of chronic HCV infection remains disproportionately high in prisons, compared to the general population [6]. These findings were similar to those of a more recent systematic review [9], in which updated prevalence estimates were lower than previously reported in most countries but still remained largely high [8, 9]. Notably, we also observed that the prevalence of anti-HCV in two Bulgarian juvenile institutions was as high as 20·5% and higher (24·7%) among studies with both adults and children. This suggests unaddressed risks and needs among younger people in prisons.

Only eight EU/EEA countries were found to have implemented the recommended universal active offer of HCV screening on an opt-out basis [17, 31], although all 26 reporting countries reported availability of HCV screening in prisons through different modalities and to varying extents. The Ministry of Health was the public authority largely responsible for prisons health in half of the countries with opt-out screening, indicating no apparent dependence on the Ministry of Health for implementation. Opt-out testing is a provider initiated service where testing is conducted unless an individual explicitly declines [32], and has been widely documented to have higher uptake than other modalities for communicable disease testing in prisons [33]. Universal active case finding, which is recommended in this setting, is known to ensure timely diagnosis and treatment to prevent the risk of further disease transmission both in and out of the prisons [17, 31]. Active case finding can be offered on a voluntary or mandatory basis, and although this was reflected in the present analysis mandatory testing is not recommended [31]. For countries reporting availability of non-mandatory testing, we assumed this implied both opt-out and opt-in modalities, where the latter refers to voluntary testing that is offered to all eligible people and the person chooses whether to have the test [31]. There was no report of on-demand screening, as this could be available within the context of active test offer, which is the recommended standard of care. Our findings differ from those of a previous survey undertaken by ECDC in 2016 [34] and the European Liver Patients’ Association (ELPA) commissioned Hep-CORE study in 2016/2017 [35]. Some plausible explanations in the conflicting findings could be changes in testing policies and practices between the surveys, different target groups and hence responses from the surveys, as well as differences between policy and actual implementation considering that patient groups in the Hep-CORE study reported absence of interventions in presence of policies.

Though modest, the available data indicated very high prevalence of IDU in prisons compared to the general population in the region. For England (United Kingdom), there were 6795 previous injectors and 7163 current injectors reported in 2016. These high numbers support the evidence that while some may stop IDU while incarcerated, many continue and others may initiate the practice while in prison [10] with implications on the continued diseases transmission as well as need for appropriate effective harm reduction interventions, including OST and NSP. The EMCDDA routinely publishes aggregated data on IDU in prisons provided annually by 30 European countries [36], and this indicator could be incorporated into the broader framework of a common monitoring system for interventions in prisons.

Data in HIPED indicated that only Spain (all prisons) and Germany (one prison) have implemented NSPs with substantially different coverage, illustrating very low coverage across the EU/EEA. In addition to these findings, both the Hep-CORE study and the European mapping of harm reduction interventions in prisons also reported implementation of NSP in prisons in Luxembourg [29, 35] whose data was not available in the HIPED. For Romania, a NSP project had been initiated but no people in prisons had been enrolled into the programme by 2017 [35], and the programme has been discontinued [29]. The prohibition of drugs in prison and safety of prison staff are some of the reasons cited for not implementing NSPs in prisons in the region [29, 37].

OST were found to be more available based on the eligibility data in the HIPED, as well as that from the EMCDDA which confirms that at least 29 of the 31 countries considered in the present analysis reported availability of OST in prisons i.e. OST could be initiated in prisons in 23 countries, and could be continued if treatment started in the community in a further 6 countries [28]. The database did not report on the actual implementation of the services, but based on the numbers on OST reported by countries against the total prisons’ populations, very few are likely to be accessing the programme except in United Kingdom. In contrast to the HIPED data and EMCDDA reports, only 11 of 20 EU/EEA countries surveyed in the Hep-CORE survey reported to have OST in all their prisons: Austria, Belgium, Croatia, France, Italy, Portugal, Romania, Slovenia, Spain, Sweden and United Kingom; including Slovakia [35] who reported having no OST according to HIPED. Besides inadequate resources, additional restrictions such as mandatory abstinence in Poland and OST initiation before incarceration in Denmark, Finland and the Netherlands [35], are barriers for the operationalisation of OST programmes in Europe [38]. Harm reduction programmes prevent acquisition of and reinfection with not only HCV but other BBVs like HIV and hepatitis B virus. This is particularly important as the burden of chronic HCV infection is as high as 94% in HIV infected people in prisons [14].

Although the HIPED database did not contain data on the availability of HCV treatment in prisons, the Hep-CORE survey conducted in 2017 found universal access in all prisons among five of the 20 EU countries included in the study: Slovakia, Slovenia, Spain, Sweden and United Kingdom; while no HCV treatment was reported to be available in Croatia and Poland [35]. In the other 13 countries, the extent of HCV treatment was reported as available in more than half of all prisons, less than half of all prisons or unknown. Another study estimated the prison population in need of treatment in France, Germany, Italy, Spain and United Kingdom (England and Wales) to be between 6000 and 9000 in 2015 [39], representing 9.3 to 11.5% of the total prisons population in these countries highlighting a large treatment gap. More recent information from the EMCDDA indicate that HCV treatment in prisons is now available in 24 European countries, but data on coverage are lacking [28]. More robust data encompassing the number of people in prisons eligible for treatment, proportion who initiate treatment, complete it and achieve viral suppression, would be central to evaluating the progress and shortfalls within the care cascade.

Despite the gaps and suboptimal coverage of key interventions in Europe, HCV elimination or near-elimination in prison is possible, as shown by few but accumulating experiences in some countries. In Iceland, the Treatment as Prevention for Hepatitis C (TraPHepC) programme, that rapidly scaled up HCV screening, access to DAAs and harm reduction services for PWID and people in prisons [3] could see the country eliminate HCV by the end of 2020 [40]. In Australia, the adoption of the ‘micro-elimination’ approach through unrestricted access to DAAs has already achieved near elimination of HCV in a prison setting [41]. This government funded project saw a remarkable reduction in viraemic prevalence of HCV from 12 to 1% in a correctional facility over 22 months, by providing voluntary testing at entry to all people in prisons, and unrestricted DAA access. Interventions targeting HCV in prison settings are cost-effective [42] and should be an integral part of any elimination strategy [43, 44].

During our analysis of the data available in the HIPED database, we encountered several limitations. We recognise that this analysis only represents a ‘snapshot’ of the situation based on incomplete and not up to date data for many of the parameters included. Not all EU/EEA countries reported to HIPED, and some countries had subnational data. The lack of an easily accessible metadata, standardised definitions and the varying prison population samples between countries further emphasise cautious interpretation of findings. The HIPED is continuously updated on a rolling basis without indication of a reference year for some parameters, and this limited the comparison of data from a temporal perspective including against published literature. Taking the aforementioned into account, discrepancies between data sources were therefore anticipated and identified. Given that this was the first data collection for the HIPED, increasing participation, data standardisation and quality control are important aspects that will be further developed over time. Aligning such monitoring programmes within the framework of the GHSS and the European action plan [18], would enable the systematic collection of standard indicators along the care pathway from prevention through to testing and treatment in line with the elimination targets.

## Conclusions

The current study reiterates the importance of a regional system to facilitate the collection and aggregation of data using standard indicators [45]. Prisons and national level monitoring could be enhanced with additional data and integrated into the European-wide monitoring system for viral hepatitis that has been rolled out by the ECDC and WHO [46]. Beyond the existing critical data gaps, our findings support previous research that the burden of IDU and HCV in prisons across the EU/EEA remains high, amidst suboptimal levels of harm reduction services, screening and treatment coverage. The population demographics, the period of detention and the DAA treatment regimen present opportunities for scaling up prison-based HCV interventions and achieving elimination goals. To realise this, a multisectoral approach and collaborations between ministries and prison departments should be adopted to intensify public health response. Engaging with the civil society will also go far in increasing awareness of the issues at hand through the effective dissemination of information.

## Data Availability

The datasets analysed during the current study are available in the SPACE I - 2018 – Council of Europe Annual Penal Statistics: Prison populations report, http://wp.unil.ch/space/files/2019/06/FinalReportSPACEI2018_190611-1.pdf; the HIPED, https://apps.who.int/gho/data/node.prisons. All_Countries?lang = en; and the ECDC Hepatitis C prevalence database, https://www.ecdc.europa.eu/en/all-topics-z/hepatitis-c/tools/hepatitis-c-prevalence-database.

## Abbreviations

anti-HCV: Antibodies against hepatitis C virus
BBV: Blood-borne virus
DAA: Direct-acting antivirals
ECDC: European Centre for Disease Prevention and Control
EEA: European Economic Area
ELPA: European Liver Patients’ Association
EMCDDA: European Monitoring Centre for Drugs and Drug Addiction
EU: European Union
GHSS: Global health sector strategy
HCV: Hepatitis C virus
HCV-RNA: Hepatitis C virus ribonucleuic acid
HIPED: Health in Prisons European Database
HIV: Human immunodeficiency virus
IDU: Injecting drug use
NSP: Needle and syringe programmes
OST: Opioid substitution therapy
PWID: People who inject drugs
UK: United Kingdom
WHO: World Health Organization
