# Correction to: Hepatitis C virus infection in EU/EEA and United Kingdom prisons: Opportunities and challenges for action

**DOI:** 10.1186/s12889-022-14317-z

**Published:** 2023-01-16

**Authors:** Aya Olivia Nakitanda, Linda Montanari, Lara Tavoschi, Antons Mozalevskis, Erika Duffell

**Affiliations:** 1 Centre for Pharmacoepidemiology, Department of Medicine Solna, Karolinska Institutet, Stockholm, Sweden; 2 European Centre for Disease Prevention and Control, Stockholm, Sweden; 3 European Monitoring Centre for Drugs and Drug Addiction, Lisbon, Portugal; 4 Department of translational research and new technologies in medicine and surgery, University of Pisa, Pisa, Italy; 5 World Health Organization Regional Office for Europe, Copenhagen, Denmark

**Correction to**: ***BMC Public Health*20, 1670 (2020).**

10.1186/s12889-020-09515-6.

The original publication of this article contained a typo in the results section of the abstract. The incorrect and correct information is shown below. The original article has been updated.

**Incorrect**

- Seroprevalence of HCV antibodies ranged from 2·3% to **82·6**% while viraemic infections ranged from 5·7% to **8·2**%, where reported.

**Correct**

- Seroprevalence of HCV antibodies ranged from 2·3% to 82·8% while viraemic infections ranged from 5·7–82%, where reported.

